# Financial conflicts of interest among editorialists in high-impact journals

**DOI:** 10.1038/bcj.2017.92

**Published:** 2017-09-15

**Authors:** V Kaestner, V Prasad

**Affiliations:** 1 Division of Hematology and Medical Oncology, Knight Cancer Institute, Oregon Health & Science University, Portland, OR, USA; 2 Department of Preventive Medicine and Public Health, Oregon Health and Science University, Portland, OR, USA; 3 Center for Health Care Ethics, Oregon Health and Science University, Portland, OR, USA

Prior studies have shown that financial relationships between editorialists and the biopharmaceutical industry are associated with increased likelihood of favorable descriptions of evidence, even when that evidence is unfavorable.^1^ How financial conflicts among editorialists should be managed—whether disclosure or recusal is necessary—has been a longstanding debate in medicine. In 1990 under editor and chief Arnold Relman, the *New England Journal of Medicine* (*NEJM*) introduced a policy of recusal for editorialists with financial ties to drugs or devices discussed in the editorial.^2^ This policy was re-affirmed in 1993 by editors Jerome Kassirer and Marcia Angell,^3^ but in 2002, citing difficulty in recruiting authors, Jeffrey Drazen and Gregory Curfman relaxed this requirement, and instituted the current policy where editorialists ‘not have any significant financial interest in any biomedical company relevant to the topics and products discussed in the article^4^’, where significant became defined as $10 000 or less in financial conflicts.^5^ Other journals, such as the *Journal of the American Medical Association* (*JAMA*) and *Annals of Internal Medicine* maintain a disclosure policy for relevant conflicts of interest for editorial writers.

Using the public disclosure of financial payments from manufactures of medical drugs or devices to physicians, we sought to identify the prevalence and amount of financial conflicts of interest among editorial writers at high impact factor journals and drug and devices discussed in the editorial.

We first chose the three highest impact factor journals in general medicine based in the United States, and as ranked by the Thompson Reuter impact factor (http://www.scimagojr.com/journalrank.php). One reviewer read all editorials published between 1 January 2016 and 31 December 2016 in the *NEJM*, *JAMA* and *Annals of Internal Medicine*. Editorial articles were included in our analysis, if they mentioned a medical drug or device, if the editorial was written by at least one author with an MD, and if that author was based in the United States. We chose these inclusion criteria because the open payments provision of the Sunshine Act applies only to US-based physicians.

The following data were collected for each article: editorial date, title, title of accompanying article, authors, drug or device studied, corresponding drug or device company.

We then used the Centers for Medicare & Medicaid Services Open Payments tool (openpaymentsdata.cms.gov) to search the names of editorial authors; general payments issued to each author from the year 2015 were recorded, the year prior to the editorial.

The search and analysis was conducted between 14 April 2017 and 1 May 2017. This study of public records did not require Institutional Review Board approval.

Out of a total 289 editorials from three high-impact medical journals in 2016, 88 (30.4%) articles discussed a drug or device and had a physician author based in the United States. Of these 88 included articles, 16 (18.2%) articles contained a conflict of interest between an author of the editorial article and a drug or device mentioned in the corresponding article. A breakdown of conflicts found in editorial articles in each journal is shown in Table 1. While 16/88 articles contained at least one conflict, several articles contained more than one conflict (Figure 1).

Of 129 editorials published *JAMA* in 2016, 23 (17.8%) discussed a drug or device and had a physician author based in the United States. Of these, 5/23 (21.7%) editorials were found to have a conflict between at least one author and the accompanying article’s drug or device. There were six authors with conflicts. Three (50%) had conflicts greater than $10 000, two had conflicts between $0–100, and one had a conflict between $100–500.

Of 106 total articles from *NEJM*, 55 (51.9%) were included in our analysis. Of those 55 editorial articles, nine (16.4%) contained a conflict between at least one author and the drug or device mentioned in the editorial’s accompanying article, according to the inclusion criteria. Of these nine conflicted articles, 12 individual conflicts were present. 3 of 12 (25%) authors had conflict greater than $10 000. 4 of 12 (33%) had conflicts between $2000–5000, one (8.3%) had conflicts between $500–1000, one (8.3%) had conflicts between $100–500, and three (25%) had conflicts between $0–100.

Of 54 total articles from *Annals of Internal Medicine* in 2016, 10 (18.5%) were included. Two of ten (20%) editorials were found to have a conflict between at least one author and the accompanying article’s drug or device. Three authors had conflicts. one (33%) over $10 000, one (33%) between $100–500, and one (33%) between $0–100.

Our investigation found that 18.2% of editorials have an author with a financial tie to the company making a drug or device discussed in the editorial. This percentage suggests that finding non-conflicted editorialists is indeed possible, as it was achieved in more than 80% of cases. Also of note, we found that 6 of those 16 (37.5%) editorials are authored by at least one person with more than the ‘significant’ threshold of $10 000 in relevant financial conflicts of interest, and this occurred in 2/9 (22%) editorials in *NEJM*. This appears to be in violation of the journal’s policies.

There are several limitations to our investigation. Our inclusion criteria excluded authors based outside the United States, as there is no database that contains their financial conflicts. Thus, our analysis only applies to US-based authors. Second, we only searched 1 year on CMS Open Payments, to ensure the relevancy of payments. Whether our results are true across years remains unknown. Unfortunately, the CMS dataset contains only 3 years (2014–2016)

As articulated 20 years ago, conflicts of interest are particularly problematic for editorial authorship, where impartiality in the adjudication of clinical research results is key, since editorials are largely based on the opinion of the authors. Moreover, empirical evidence suggests that the stance taken in an editorial is influenced by financial ties to the sponsor, with payment linked to more favorable views.^1, 6^ For these reasons, it may be an unfair burden to ask readers to tease out the relationships between conflicts of interest and expert opinions, and weigh these in consideration of the editorial.

## Figures and Tables

**Figure 1:**
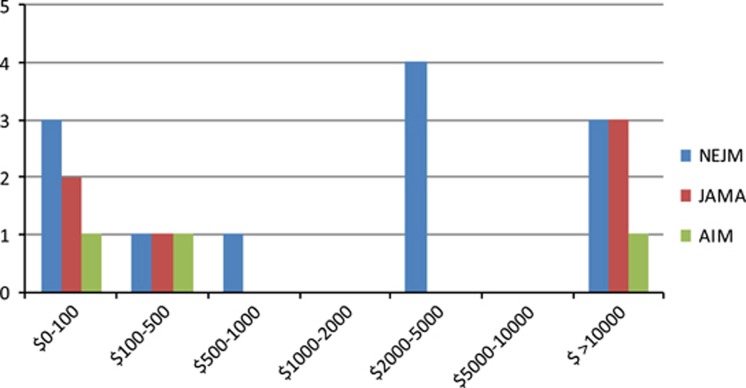
Number of Editorialists with Conflict, by Value of Conflict.

**Table 1:** Breakdown of editorials and conflicts

*NEJM*	*JAMA*	*Annals of Internal Medicine*
106 total editorials	129 total editorials	54 total editorials
55 included	23 included	10 included
12 conflicts, 9 people	6 conflicts, 6 people	3 conflicts, 2 people
9 conflicted editorials, 9/55 (17.6%)	5 conflicted editorials, 5/23 (21.7%)	2 conflicted editorials, 2/10, (20%)
